# DL-3-n-butylphthalide promotes hippocampal neurogenesis and reduces mossy fiber sprouting in chronic temporal lobe epilepsy rats

**DOI:** 10.1186/s12883-021-02516-x

**Published:** 2022-01-03

**Authors:** Shanshan Zhao, Fangxi Liu, Wei Shi, Jialu Wang, Zhike Zhou, Xiaoqian Zhang

**Affiliations:** 1 Department of Neurology, The First Affiliated Hospital, China Medical University, Shenyang, 110001 Liaoning China; 2 Department of Neurology, Tacheng District People’s Hospital, Tacheng, 834700 Xinjiang China; 3 Department of Geriatrics, The First Affiliated Hospital, China Medical University, Shenyang, 110001 Liaoning China

**Keywords:** DL-3-n-butylphthalide, Temporal lobe epilepsy, Neurogenesis, Cognition

## Abstract

**Background:**

A decrease in hippocampal neurogenesis is considered an important cause of cognitive impairment, while changes in mossy fiber sprouting are closely related to development of spontaneous recurrent seizures in chronic temporal lobe epilepsy (TLE). Racemic l-3-n-butylphthalide (DL-NBP) can alleviate cognitive impairment in ischemic stroke and Alzheimer’s disease by promoting neurogenesis. DL-NBP treatment can also improve cognitive function and reduce seizure incidence in chronic epileptic mice. However, the mechanisms of action of DL-NBP remain unclear. The aim of the present study was to examine the effects of DL-NBP on mossy fiber sprouting, hippocampal neurogenesis, spontaneous epileptic seizures, and cognitive functioning in the chronic phase of TLE.

**Methods:**

Nissl staining was used to evaluate hippocampal injury, while immunofluorescent staining was used to analyze hippocampal neurogenesis. The duration of spontaneous seizures was measured by electroencephalography. The Morris water maze was used to evaluate cognitive function. Timm staining was used to assess mossy fiber sprouting.

**Results:**

TLE animals showed reduced proliferation of newborn neurons, cognitive dysfunction, and spontaneous seizures. Treatment with DL-NBP after TLE increased the proliferation and survival of newborn neurons in the dentate gyrus, reversed the neural loss in the hippocampus, alleviated cognitive impairments, and decreased mossy fiber sprouting and long-term spontaneous seizure activity.

**Conclusions:**

We provided pathophysiological and morphological evidence that DL-NBP might be a useful therapeutic for the treatment of TLE.

## Background

Temporal lobe epilepsy (TLE) is the most common type of intractable epilepsy, the major characteristic of which is spontaneous recurrent seizures (SRS). Hippocampal injury and reduced neurogenesis caused by recurrent or uncontrolled seizures are related to cognitive dysfunction and can markedly affect the quality of life of patients [1–3]. Furthermore, approximately 33% of epilepsy patients suffer from pharmaco-resistance, which represents a major challenge to successful clinical therapy [4]. Therefore, finding effective drugs to reduce SRS and improve cognitive dysfunction associated with epilepsy is of high importance.

L-3-n-butylphthalide (NBP) is the extraction of Apium graveolens Linn seeds (Chinese celery). One isomer of NBP, DL-NBP, has been synthesized and is currently in clinical trials for treatment of acute ischemic stroke. Recent studies have shown that DL-NBP improves cognitive function in traumatic brain injury, ischemic stroke, and Alzheimer’s disease, likely by reducing neural loss and promoting neuronal regeneration [5–8]. However, few studies addressed whether and how DL-NBP treatment affected neurobehavioral outcomes and pathophysiological changes in the experimental TLE model chronic stage. One study confirmed that DL-NBP could improve cognitive function and alleviate seizure severity in mouse with chronic epilepsy [9]. They proved that DL-NBP treatment reduced the loss of hippocampal neurons in mice with chronic epilepsy. This suggests that DL-NBP may serve as a new therapeutic strategy for preventing cognitive deterioration by reducing neural loss. Nevertheless, the effects of DL-NBP treatment on hippocampal neurogenesis and the mechanisms of reducing seizure incidence by DL-NBP treatment in TLE model are unknown.

In the present study, we examined effects of DL-NBP on mossy fiber sprouting, hippocampal neurogenesis, spontaneous epileptic seizures, and cognitive function in the chronic phase of TLE in rats.

## Methods

### Experimental groups

Adult male Wistar rats (220–250 g) were divided randomly into sham-operated (SHAM; *n* = 20), epilepsy (EP; *n* = 20), and epilepsy plus DL-NBP treatment (EP+NBP; *n* = 20) groups. Each rat was housed separately during postoperative status epileptics, and then each group was fed in separate cages (five rats per cage) after status epileptics. All animals were housed in standard humidity and temperature conditions in a 12 h light/dark cycle, with *ad libitum* access to food and water. All experimental procedures were approved by the Animal Care and Use Committee of China Medical University. All animal experiments complied with the Animal Research: Reporting of In Vivo Experiments (ARRIVE) guidelines and were carried out in accordance with the Regulations for the Administration of Affairs Concerning Experimental Animals approved by the State Council of People’s Republic of China.

### TLE model

TLE model animals were established by injection of kainic acid (KA) into the right lateral ventricle [10]. Rats were anesthetized using a mixture of 70% nitrous oxide and 3% isoflurane in 30% oxygen, and maintained with 1.5% isoflurane. A solution of 0.4 μg/μL KA (2 μL total volume) in sterile saline was injected into the right ventricle using a Hamilton microsyringe (stereotactic coordinates: −1.5 mm mediolateral, −4.0 mm dorsoventral, and +0.8 mm anteroposterior; depth determined from the cerebral surface) and an infusion pump (0.5 μL/min). The needle was left in place for 5 min after the injection and then removed slowly. The first seizures began at 15–30 min after KA injection. The animals were removed the stereotaxic frame and then allowed to recover from anesthesia. A total of 47 rats received KA injection, with seven deaths (mortality = 14.9%).

Seizure severity was graded using five levels according to the Racine scoring system. Grade IV animals with more severe attack severity and animals with an attack duration >2 h were used in this study. After approximately 2 weeks of recovery, animals developed focal SRS accompanied by interictal spikes and waves (chronic phase). SHAM animals were injected with normal saline using the same procedures described above, with no deaths.

### DL-NBP administration

The purity of DL-NBP was >99.5% (CSPC-NBP Pharmaceutical Co., Ltd., Hebei Province, China). DL-NBP was diluted to 7 mg/mL in vegetable oil and administered orally at a dose of 70 mg/kg/day from the 7^th^ day post-surgery for 2 weeks.

### Water maze test

Rats were subjected to the water maze test on days 31–33 after seizure initiation. As we reported, the match-to-place version was used to evaluate spatial learning [10]. On the last day of testing (postoperative day 33), the 60 s probe test without a platform was performed to estimate memory of the platform position (number of passes through the previous platform location).

### Electroencephalograph

Electroencephalograph (EEG) was recorded from rats in the EP group and the EP+NBP group on days 34–36 after surgery. Intracranial EEG electrodes were placed into the right hippocampus (*n* = 5 per group). The electrodes were embedded within the CA3 region of the right hippocampus (2.4 mm lateral to midline, 3.4 mm ventral to dura, 3.8 mm posterior to bregma) and the regio nasalis using coordinates derived from the Paxinos and Watson atlas. After two days of recovery, the total duration of spontaneous EEG seizures over 2 h was recorded from freely moving animals. Digital acquisition software (LabScribe2; iWorx, USA) was used to record the EEG signals, which were stored for off-line analysis.

### Tissue preparation

On day 37 of recovery, animals were perfused transcardially with 4% paraformaldehyde in phosphate-Buffered Saline. The tissues were post-fixed and stored in 30% sucrose solution at 4 °C. The brains were cut into coronal sections from 2.8 to 4.3 mm posterior to bregma (30 μm thick) on a cryotome (Thermo Electron, Waltham, MA, USA), when they were in the bottom. For each animal, five from every series of ten sections were used for immunofluorescent staining.

### Immunofluorescent staining

Brain sections were incubated with primary antibodies against guinea pig anti-doublecortin (DCX; 1:400; Abcam) at 4 °C overnight, followed by incubation in Alexa Fluor 488 goat anti-rabbit secondary antibodies (1:200; Invitrogen, USA) for 2 h. Images were collected by microscopy (Olympus, Japan) using a ×20 or ×40 objective. The number of DCX-positive cells was counted using NIH ImageJ software (https://imagej.nih.gov/ij).

### Nissl staining

Brain tissues were fixed with paraffin and then cut into a series of coronal sections (10 μm thick). Brain sections were then incubated in toluidine blue solution for 20 min at 56 °C, quickly washed in distilled water, incubated in 95% alcohol for 5 min, dehydrated in increasing alcohol concentrations for 5 min, cleared for 5 min with xylene, and then mounted in neutral gum solution. The number of CA3 pyramidal neurons in the hippocampus (per 1 mm^2^) in both cerebral hemispheres was counted by microscopy with a high magnification objective.

### Timm staining

Animals in the three groups (*n* = 5 per group) were deeply anesthetized and then perfused intracardially with sodium sulfide solution (1.2% Na_2_S·9H_2_0, 1% NaH_2_PO_4_·H_2_0) and normal saline. The brains were then removed and post-fixed in paraformaldehyde solution. Coronal sections were stained with Timm staining solution for 90 min at room temperature. Images were taken using an Olympus BX51 microscope.

### Statistics

All statistical analyses were performed using statistical software (SPSS v25). Water maze data were analyzed by repeated-measures or one-way analysis of variance. The remaining data were analyzed using the Kruskal–Wallis or Mann–Whitney U non-parametric tests. All data are presented as mean ± standard error of the mean. Significance was set at *P* < 0.05.

## Results

### DL-NBP treatment promotes neurogenesis in the dentate gyrus

**Fig. 1:**
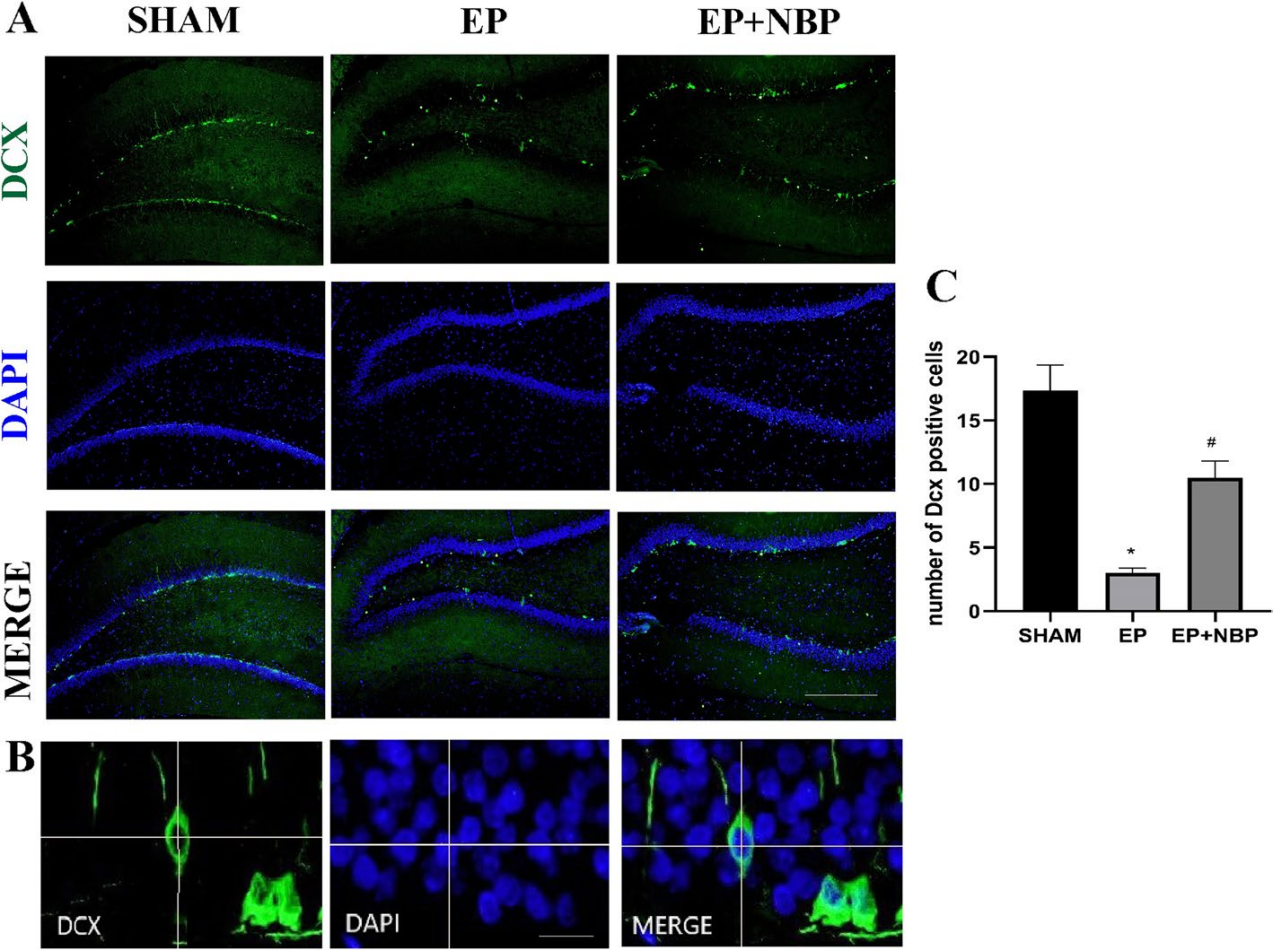
Proliferation of newborn neurons in the dentate gyrus (DG). **a** Newborn cells were labeled with both diamidino phenylindole (DAPI; blue) and the specific immature granule cell-maker doublecortin (DCX; green). **b** Reconstructed orthogonal images(x–z and y–z planes) displaying DCX or DAPI immunoreactivity separately or as a merged image. **c** The number of DCX-positive cells in the EP group was significantly less than in the SHAM group (*P < 0.05 vs. SHAM). DL-NBP treatment significantly increased cell proliferation (#*P* < 0.05 vs. EP). *n* = 5 per group. Scale bars: **a** 50 μm; **b** 20 μm

Immunofluorescent staining of DCX-positive neuroblasts was performed to examine whether DL-NBP treatment could regulate neurogenesis in the dentate gyrus (DG) (Fig. 1). At the chronic stage of epilepsy, the number of hippocampal DCX-positive cells in the EP group was significantly reduced compared with the SHAM group (3.0 ± 0.4 vs. 17.3 ± 2.0, respectively; *P* < 0.05). DL-NBP treatment was associated with a significant increase in the number of DCX-positive newborn neurons compared with the EP group (10.5 ± 1.3 vs. 3.0 ± 0.4, respectively; *P* < 0.05). These data indicate that DL-NBP treatment increased the number of newborn neurons in the DG following KA-induced epilepsy.

### DL-NBP treatment reduces hippocampal damage

**Fig. 2:**
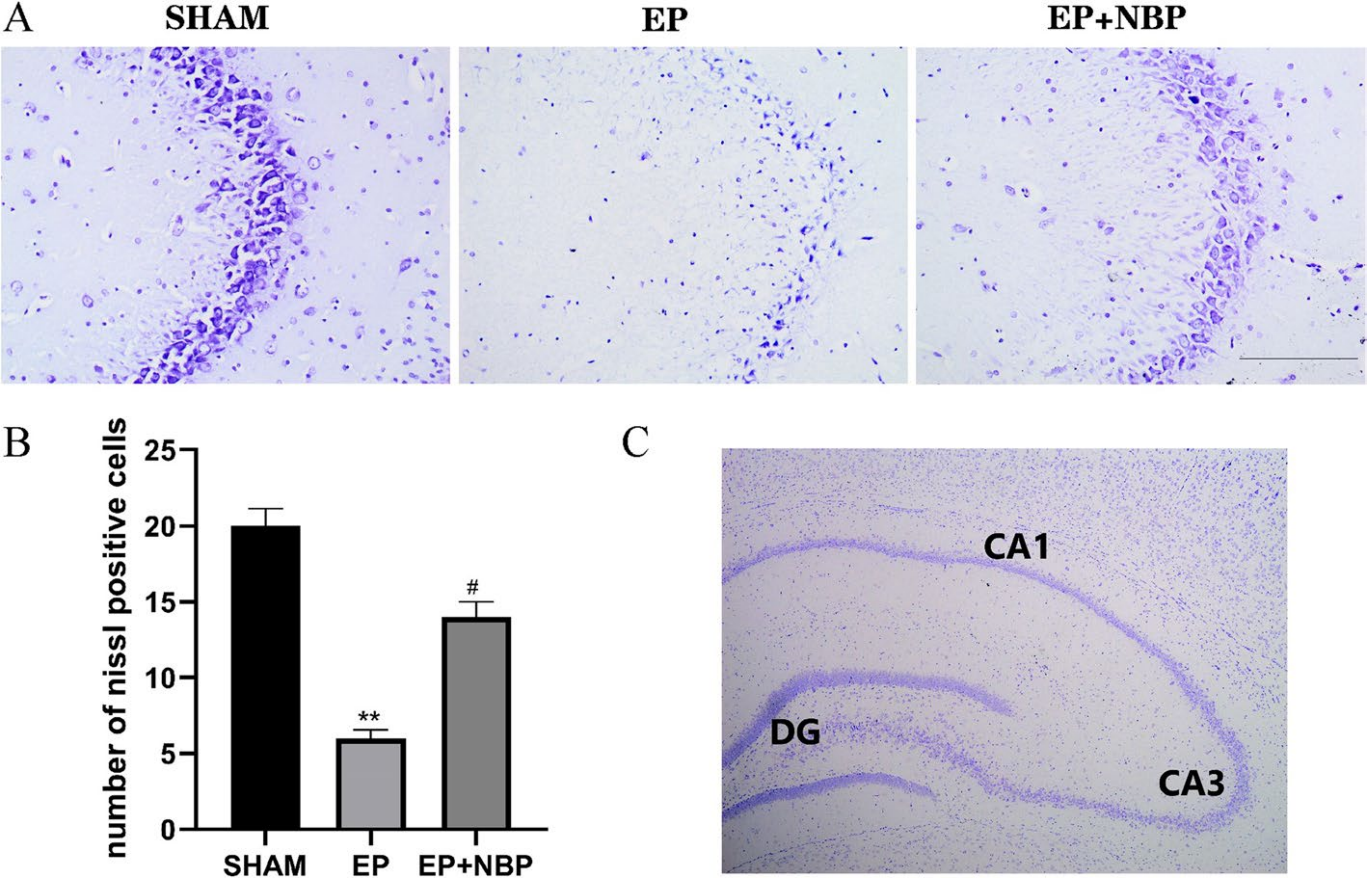
Numbers of neurons in the CA3 region of the hippocampus. **a** Microscopy image of the CA3 region (×20 magnification). **b** There was a significant loss of CA3 neurons in the EP group compared with the control (SHAM) group, while DL-NBP treatment increased the number of CA3 neurons. **c** Microscopy image of the hippocampus (×4 magnification). (**P < 0.01 vs. SHAM; #*P* < 0.05 vs. EP; *n* = 5 per group). Scale bars: 50 μm. *DG* dentate gyrus

Next, we performed Nissl staining to evaluate hippocampal neuronal damage (Fig. 2). There was a significant reduction in the number of CA3 neurons in the EP group compared with the SHAM group (6.0 ± 0.6 vs. 20.0 ± 1.2, respectively; *P* < 0.01). DL-NBP treatment was associated with a significant increase in the number of Nissl-positive neurons in the CA3 compared with the EP group (14.0 ± 1.0 vs. 6.0 ± 0.6, respectively; *P* < 0.05). These data suggest that DL-NBP treatment attenuated neuronal loss caused by KA-induced epilepsy.

### DL-NBP treatment decreases the frequency and duration of spontaneous EEG seizures

**Fig. 3:**
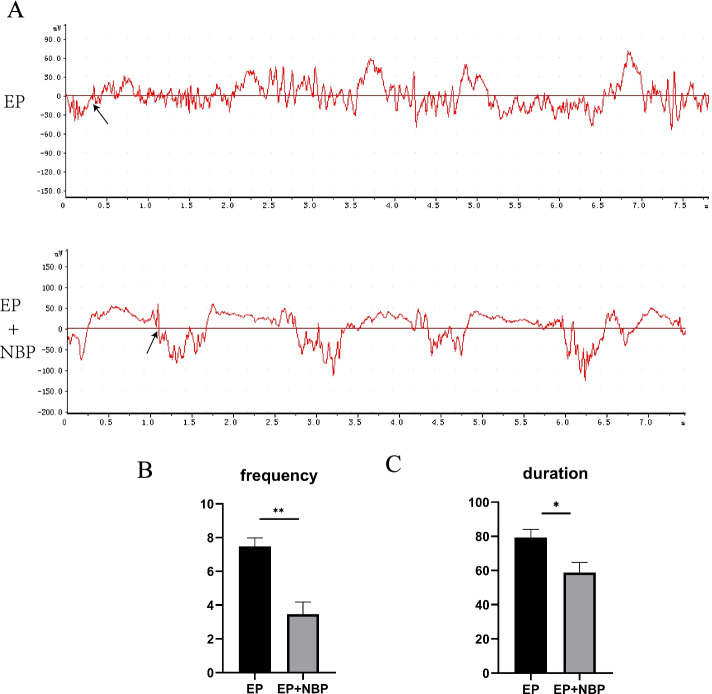
Spontaneous EEG seizures in the dentate gyrus (DG). **a** Image displays a representative example of rhythmic epileptiform EEG activity in the EP and EP+NBP groups. **b, c** Post-seizure treatment with DL-NBP decreased the duration of spontaneous EEG seizures (**P < 0.01 vs. EP; *P < 0.05 vs. EP; *n* = 5 per group). Black arrows indicate the start of the seizures

Spontaneous EEG seizures were assessed on day 36 after surgery (Fig. 3). No spontaneous seizures were observed during EEG recording in the SHAM group. Compared with the EP group, DL-NBP treatment significantly reduced the frequency (7.5 ± 0.5 vs. 3.5 ± 0.7 seizures/2 h, respectively; *P* < 0.01) and duration (79.3 ± 4.8s vs. 58.8 ± 5.9s /2 h, respectively; *P* < 0.05) of spontaneous EEG seizures following KA-induced epilepsy.

### DL-NBP treatment decreases mossy fiber sprouting

**Fig. 4:**
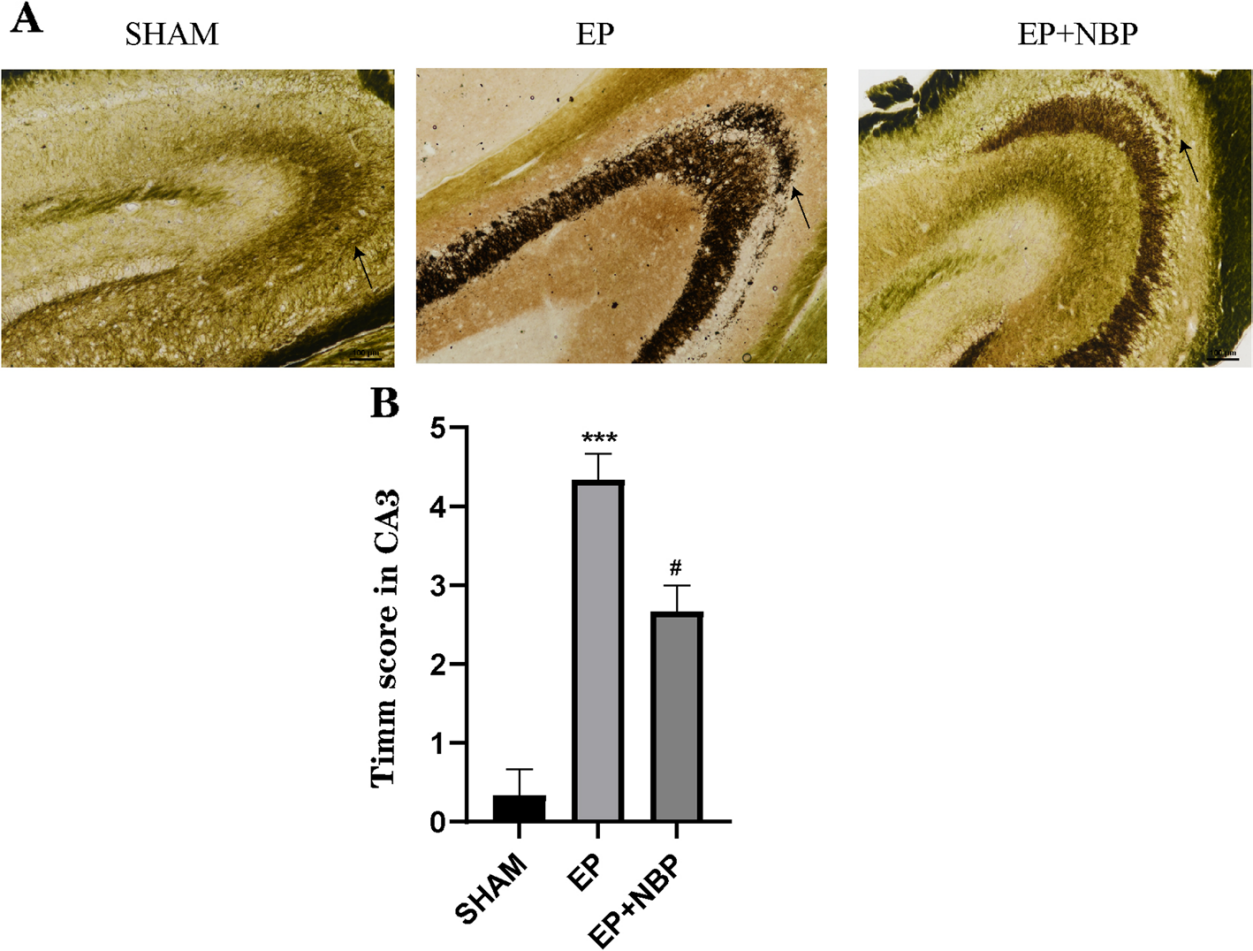
Timm staining in the CA3. Timm scores were significantly increased in the EP group compared with the control group (***P < 0.001 vs. SHAM) and significantly decreased after DL-NBP treatment (#*P* < 0.05 vs. EP; *n* = 5 per group). Black arrows indicate the mossy fiber sprouting

Mossy fiber sprouting in the hippocampus was evaluated using Timm staining (Fig. 4). Compared with the SHAM group, there was a significant increase in the Timm score after seizure onset in the EP group (0.3 ± 0.3 vs. 4.3 ± 0.3, respectively; *P* < 0.001), which was significantly reduced following DL-NBP treatment (4.3 ± 0.3 vs. 2.7 ± 0.3, respectively; *P* < 0.05).

### DL-NBP improves cognitive function in rats with TLE

**Fig. 5:**
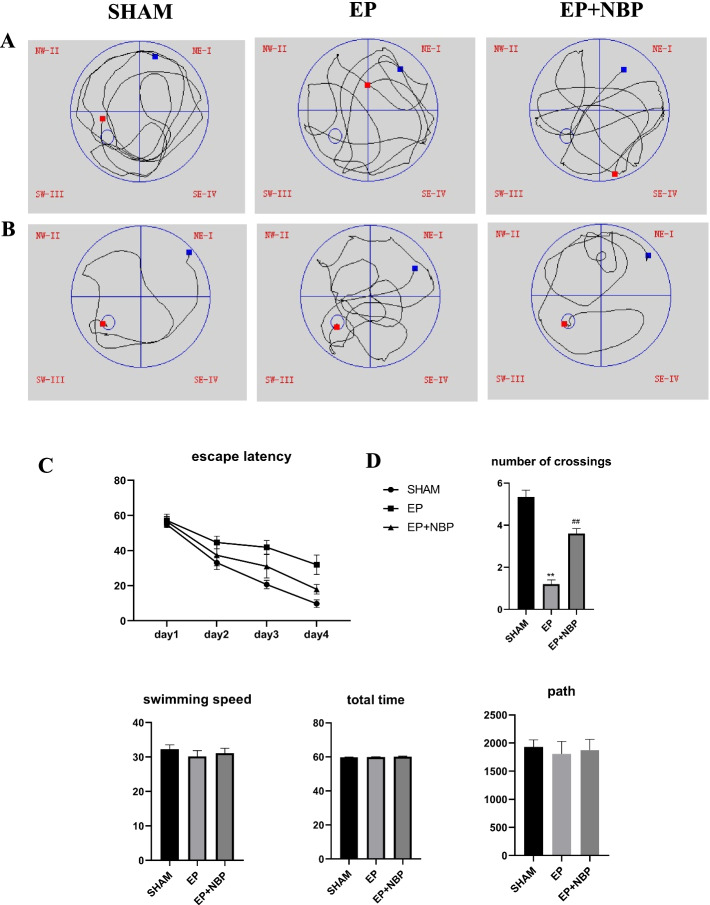
Morris water maze performance. **a** Representative swim paths in the probe trial test. **b** Representative swim paths in the water maze test. **c** Escape latency to the platform during the match trial in the water maze test. **d** Number of crossings in the probe trial test. Blue square indicates starting points. Red square indicates finishing points. *P < 0.05 vs. SHAM, **P < 0.05 vs. SHAM, #*P* < 0.05 vs. EP (*n* = 15 per group)

We performed the Morris water maze test to evaluate changes in cognitive function (Fig. 5). Compared with the SHAM group, rats in the EP group showed a significant increase in escape latency (F[2, 4] = 35.581, *P* = 0.041), while DL-NBP treatment was associated with a significant decrease in the escape latency compared with the EP group (F[2, 4] = 35.581, *P* = 0.016). There were no differences in swimming speed, the total swimming path, and the total swimming time between the different groups. In the probe test, animals in the EP group showed a significant reduction in the number of crossings compared with the SHAM group (1.2 ± 0.2 vs. 5.3 ± 0.3, respectively; *P* < 0.01). Treatment with DL-NBP was associated with a significant increase in the number of crossings compared with the EP group (3.6 ± 0.25 vs. 1.2 ± 0.2, respectively; *P* < 0.01).

## Discussion

In the present study, adult rats were randomly assigned to control rats, rats with epilepsy, or rats with epilepsy treated by DL-NBP for 30 days. The key findings of the study were that, in the chronic phase of seizures in TLE rats, DL-NBP treatment could reverse neural loss and reduce mossy fiber sprouting in the hippocampus and increase neurogenesis in the DG. Furthermore, DL-NBP treatment was associated with an improvement in spatial learning cognitive function and a decrease in long-term seizure activity.

Chronic epilepsy can cause changes in the hippocampal microenvironment [11], which can affect neuronal survival and ultimately result in neuronal loss in the DG [11]. Furthermore, several TLE studies have reported that neurogenesis in the DG can rapidly increase after acute seizures [10], but decrease in the chronic phase [12, 13]. This decrease in hippocampal neurogenesis in chronic TLE is caused by decreased neuronal proliferation and differentiation of newborn cells [14]. The continuous production of hippocampal newborn neurons and their integration with existing neural circuits support learning and memory in the DG [15]. The lack of existing neurons and insufficient survival rate of newborn neurons make it difficult to maintain the integrity of neural circuits, which is critical for normal cognitive function [16]. In the present study, we used the Morris water maze test of spatial learning and memory to assess cognitive function. Nevertheless, follow-up studies should consider other hippocampal-dependent tasks, including the contextual fear conditioning task and novel object recognition task, to provide a more comprehensive analysis of cognitive function.

DL-NBP is a multiple growth factor activator that shows neuroprotective effects in ischemia-reperfusion injury [17] and chronic cerebral hypoperfusion [8]. The mechanisms of action include regulation of apoptosis and autophagy-related protein expression, and activation of Akt signaling [18]. There are also evidences that DL-NBP can reduce neuronal damage in various central nervous system diseases, including ischemic stroke, traumatic brain injury, and Alzheimer’s disease [6, 7, 19]. Furthermore, NBP treatment can reduce cognitive impairment by accelerating neurogenesis and restoring synaptic plasticity [9, 20, 21]. Currently, DL-NBP is widely used for treatment of ischemic stroke patients in China. The guidelines for the prevention and treatment of stroke in China (2021 edition) clearly state that DL-NBP is effective in improving cognitive function and overall function in patients with vascular cognitive impairment and has a good safety and tolerability profile. However, DL-NBP is not currently used clinically in patients with epilepsy in China and is unavailable in other countries.

With respect to chronic epilepsy, DL-NBP was found to increase levels of brain-derived neurotrophic factor, neuroprotective factor, and Klotho level [9]. However, the effect of DL-NBP on neurogenesis remains unclear. Consistent with a previous report [9], we found that DL-NBP treatment reversed cognitive impairment and reduced neural loss in the hippocampus following TLE. These findings suggest that DL-NBP may be protective against neuronal death following TLE. We also found that DL-NBP significantly increased the number of hippocampal DCX-positive cells in the chronic stage of epilepsy. These findings suggest that DL-NBP can promote neurogenesis following TLE. Overall, our data suggest that DL-NBP may improve cognitive function following TLE by increasing DG neurogenesis and reducing hippocampal damage. Further studies are required to determine the specific pathways by which Dl-NBP exerts its anti-apoptotic actions.

Epilepsy is the result of an imbalance between excitatory and inhibitory neuronal activity. Recurrent seizures are a major cause of cognitive impairment [22]. Interestingly, DL-NBP treatment was reported to regulate the balance between excitatory and inhibitory neuronal activity, thus reducing seizure severity [9]. Consistent with these findings, we found that DL-NBP treatment reduced the frequency and duration of spontaneous EEG seizures. Mossy fiber sprouting is considered a major feature of aberrant tissue remodeling after neural loss caused by epilepsy in both humans and animal models [23, 24]. Recent studies also suggest that reduced mossy fiber sprouting is a potential mechanism of action of several anti-epilepsy therapies, which may reduce hippocampal neuronal injury [25–27]. Importantly, we found new evidence for a lower Timm score in NBP-treated epilepsy rats, which is likely associated with the reduced frequency of seizure onset. Thus, DL-NBP may decrease the frequency of seizures by reducing mossy fiber sprouting in chronic TLE rats.

## Conclusions

We found that DL-NBP treatment can improve cognitive function in adult rats after TLE, as well as inhibit long-term seizure activity, reverse neural loss, increase neurogenesis, and reduce mossy fiber sprouting. Thus, DL-NBP may be a useful therapeutic agent for TLE. Nevertheless, direct evidence and specific mechanism are needed to provide in future research, that is, enhanced neurogenesis and decreased mossy fibers are the reason for the antiepileptic effect of DL-NBP in TLE.

## Data Availability

The datasets used and/or analysed during the current study are available from the corresponding author on reasonable request.

## Abbreviations

TLE: Temporal lobe epilepsy
NBP: L-3-n-butylphthalide
DL-NBP: DL-3-n-butylphthalide
SRS: Spontaneous recurrent seizures
KA: Kainic acid
EEG: Electroencephalograph
DCX: Doublecortin
DG: Dentate gyrus
